# Combination of the Napkin and the Rubber Dam

**Published:** 1885-01

**Authors:** B. M. Wilkeson

**Affiliations:** Baltimore, Md.


### COMBINATION OF THE NAPKIN AND THE RUBBER DAM.

BY B. M. WILKESON, D. D. S., M. D., BALTIMORE, MD.

For filling the superior teeth, when it is not desirable to use the rubber dam in the usual way, but it is important to keep the napkin dry, I find it an admirable plan to place the napkin upon a sheet of rubber dam, with the latter projecting about half an inch beyond the edge of the former. I then pass each under the teeth to be filled, and hold them in the same manner as when the napkin alone is used. In this way the napkin will be kept thoroughly dry, which will enable the dentist to perform his operations with great satisfaction.

A little experience in the use of this combination will prove particularly valuable to those who use soft gold and amalgam, as well as all other filling materials. The advantages which I have derived in this method are: It saves much valuable time that would otherwise be spent in adjusting the dam in the usual manner; it protects the napkin from moisture, which would be absorbed from the secretions in the lower part of the mouth; the napkin throws more light into the cavities to be filled than would the dam alone, and the gold is not so liable to drag on the linen as on the rubber; the two are more readily held in position than would the rubber alone.

This combination may not be new to all, nevertheless I have never heard of any other operator using it; therefore, as far as I know, it is original with me, and I publish the discovery, not as a revolution, but in order that others may reap the benefit.

